# Improved Optimization Strategy Based on Region Division for Collaborative Multi-Agent Coverage Path Planning

**DOI:** 10.3390/s23073596

**Published:** 2023-03-30

**Authors:** Yijie Qin, Lei Fu, Dingxin He, Zhiwei Liu

**Affiliations:** School of Artificial Intelligence and Automation, Huazhong University of Science and Technology, Wuhan 430070, China

**Keywords:** mCPP, multi-agent system, complete coverage, minimum coverage paths

## Abstract

In this paper, we investigate the algorithms for traversal exploration and path coverage of target regions using multiple agents, enabling the efficient deployment of a set of agents to cover a complex region. First, the original multi-agent path planning problem (mCPP) is transformed into several single-agent sub-problems, by dividing the target region into multiple balanced sub-regions, which reduces the explosive combinatorial complexity; subsequently, closed-loop paths are planned in each sub-region by the rapidly exploring random trees (RRT) algorithm to ensure continuous exploration and repeated visits to each node of the target region. On this basis, we also propose two improvements: for the corner case of narrow regions, the use of geodesic distance is proposed to replace the Eulerian distance in Voronoi partitioning, and the iterations for balanced partitioning can be reduced by more than one order of magnitude; the Dijkstra algorithm is introduced to assign a smaller weight to the path cost when the geodesic direction changes, which makes the region division more “cohesive”, thus greatly reducing the number of turns in the path and making it more robust. The final optimization algorithm ensures the following characteristics: complete coverage of the target area, wide applicability of multiple area shapes, reasonable distribution of exploration tasks, minimum average waiting time, and sustainable exploration without any preparation phase.

## 1. Introduction

Autonomous unmanned systems, which can replace humans in dangerous or inaccessible workspaces, are currently a popular research topic, and many results have been achieved in related control synergy methods. A fundamental problem in autonomous unmanned systems is to determine an optimal path that passes through all points of a given region of interest, while avoiding specific subregions (e.g., obstacles, no-entry zones, etc.). In the scientific literature, this problem is often referred to as the coverage path planning problem (CPP), but it can also be referred to as sweeping, complete geographic search, area patrol, etc. Typical applications are the work of detection and rescue cameras [1,2], ground penetrating synthetic aperture radar (GPSAR) surveys of suspected hazardous areas [3]. There are also wide applications in daily life, such as vacuum cleaning robots, painter robots, lawn mowers, automatic harvesters, window cleaners, and inspection of complex structures, among others.

Nowadays, multi-agent systems improve the performance of individual agents through information sharing and communication collaboration, such as task execution efficiency, robustness, flexibility, and fault tolerance, resulting in distributed decision-making, formation control, area coverage, and other related applications. The mCPP problem discussed in this paper, aims to plan optimal paths for each agent. The paths will form a path coverage of the global target region for the purpose of protecting or supervising this region, while minimizing the total coverage time.

Although numerous solutions for generating coverage paths in general polygonal environments already exist, these implementations are either not publicly available, computationally unsuitable for large and complex environments, have realistic application flaws in the planned paths, or lack complete system integration. In this paper, we propose a strategy that can quickly generate full-coverage paths for multiple agents in non-convex regions, based on recent multi-agent traversal algorithms [4,5,6], extending the coordinate multi-agent exploration strategy introduced in [7,8]. The strategy improves the ability to cope with narrow spaces and near-closed regions (which are necessary in various applications, such as search and rescue) and greatly reduces iterations for the corner cases mentioned above. Meanwhile a more rational sub-region assignment greatly reduces the number of turns in the paths. The final solution we propose takes a general polygon as input and distributes the target region into multiple subregions, considering the initial location and task load of the multiple agents. A closed trajectory is then computed for each agent, which ensures that each point in the region is visited cyclically with the same frequency. The contributions of this work are listed below:

1. In order to cope with the corner case of narrow or near-enclosed areas, geodesic distances are used for initial area assignment, which greatly improves the efficiency of the next iterations;
2. Dijkstra algorithm is applied to dynamically assign weights in calculating geodesic distances, optimizing the shape of the subregion, reducing path turns, and improving energy efficiency;
3. The characteristics of paths generated by RRT-based methods are improved by selecting the most suitable among different weight schemes when building spanning trees.
4. Using a complex indoor scenario as an example, the full-flow solution of this paper is applied for simulation implementation, and the performance of the planned path scheme is quantified and evaluated.

This paper is organized as follows. Section 2 reviews the state-of-the-art in multi-agent regional exploration and compares different algorithms proposed by various scholars, further clarifying our representation of the regional path covering problem, while further clarifying our representation of the mCPP problem. Section 3 provides a detailed description of the strategy’s implementation process in terms of environment description, region partitioning, and sub-region path calculation. Section 4 describes the experimental setup and task planning process for a case of an indoor complex environment, while designing and quantifying the path indicators of mCPP. In Section 5, the conclusions drawn from the comparative evaluation are summarized and future work is proposed.

## 2. Related Works

Coverage path planning in a known environment, also known as repetitive sweeping or region traversal, can be formulated in detail as follows: given a target area and a sensor model, a feasible path is planned for mobile robot that provides complete actual access to the target area while minimizing some cost metric (usually path length or maximum waiting time), under the premise of the motion model and spatial constraints [9]. The basic algorithms for solving the CPP problem in two-dimensional polygonal regions are usually based on approximate or precise cell decomposition [10,11], which are closely related to the Art Gallery Problem (AGP) and the Traveling Salesman Problem (TSP) [9]. Both of the problems are NP-hard [12] and heuristics have been developed to cope with high-dimensional problems.

One of the simplest exact unit decomposition techniques that can generate complete coverage paths is the trapezoidal decomposition method [13,14]. For the specific path covering each unit, the “Z” shape or “mowing the lawn” back-and-forth motion is typically used. Franco and Buttazzo [15] proposed a method to enable cyclic exploration by making the number of stripes even, allowing the returned path to be used as a scan path. This method divides the free space (i.e., the space without obstacles) into non-overlapping regions. However, as the number of units increases, the total coverage path becomes longer in order to connect the paths of each unit. The boustrophedon decomposition proposed by Choset and Pignon [16,17] effectively reduces the number of units compared to the former, while the units are not limited to convex regions. Compared to the aforementioned methods, the Morse-based unit decomposition [18] has the advantage of handling non-polygonal obstacles.

The methods mentioned above can be classified as offline methods. Acar and Choset [19,20] have proposed an online complete coverage method, which performs coverage of the planar space through the detection within the sensing range. In addition, Wong [21,22,23] have proposed landmark-based topological coverage strategies, which can explore the boundary of the current unit and update the topological map, achieving online complete coverage. The key technologies for online complete coverage lie in the acquisition of two-dimensional and three-dimensional point data, obstacle detection, recognition and avoidance, and data transmission and sharing. The latest advances in machine vision theory and its application in robot navigation are presented in [24], which provides valuable theoretical and practical guidance for the design and application of online methods.

In general, single-agent CPP, as an early control problem, has mature strategies available after years of research and validation. The use of multiple agents for regional complete coverage is a recent challenge for researchers. Table 1 briefly introduces the recent methods of mCPP problem, and compares and summarizes the current state-of-the-art techniques and some key features of multi-agent coverage path planning strategies. The symbol “√” indicates that the algorithm supports this advantageous feature, while the symbol “×” implies that the algorithm does not support this feature.

Puig [25] utilized a K-Means clustering method to assign required sub-regions. Based on the initial location of the agents, each agent is assigned to its corresponding working area, reducing the variance of the minimum waiting time. However, this method cannot fundamentally eliminate the time/cost of reaching these sub-regions for the agents to reach their corresponding sub-regions.

Sariel [26] treats the multi-agent exploration problem as an extension of the multiple TSP problem and proposes a real-time single-item auction-based allocation strategy. This behavior is similar to greedy and clustering methods, and robots tend to choose distant targets and tend to separate. However, the process of robots choosing their target domains is not efficient enough, and the target domains of each robot may be highly imbalanced.

Maza [27] proposed a mCPP method for UAVs. The algorithm assigns small and exclusive sub-regions to each UAV and calculates a minimized reciprocal coverage path within their exclusive sub-regions. However, it can only handle convex polygonal regions and does not support the presence of obstacles within it. The method also assumes an unrealistic condition, that the robots are initially positioned on the boundary of the target region.

In unknown environments, intelligent swarms require dynamic mapping using the sensors of each agent, while data sharing and real-time communication among robotic agents typically require a distributed algorithm. Recent research [29,30] has proposed an effective method to address navigation and communication optimization problems for robotic swarms operating in densely obstructed areas. These studies also provide optimization solutions for multi-agent collaborative mapping, navigation, and sensor data fusion. These findings have significant implications for achieving complete coverage of multiple agents in an online environment.

The focus of many studies on the mCPP problem has been on the task partitioning, while a simple “Z” pattern of coverage path is adopted for the decomposed units. Li [31] and Torres [32] present methods to optimize the scanning direction instead of using a fixed direction for all units, which can minimize the number of unit traversals and turns on the coverage paths. However, it comes at the cost of an increase in computational complexity when solving for the optimal path of the connected graph. The X-STC algorithm based on spanning tree, proposed by Guruprasad [33], divides the workspace into non-overlapping units and generates a path around the spanning tree, which ensures that every point in the grid is covered exactly once. This circular path has the advantage of avoiding redundant motion.

The grid-based method uses grid cells of uniform resolution for a decomposed representation of the target region. This representation using grids was first proposed by Moravec and Elfes [5]. However, the memory usage of the grid map grows exponentially as the region expands. This is because the resolution is fixed regardless of the complexity of the environment [34].

The literature [8,28] handles the multi-agent problem by reducing the mCPP problem to single-robot CPP problems. This is achieved by dividing the entire region into equal and exclusive subregions. Although the authors state that the method greatly reduces the complexity compared to other state-of-the-art methods when a solution exists, the illustration used to show the iterative process is an ideal convex region without obstacles. We found that the method is difficult to converge to a solution when the target region is a narrow area or a near-closed area. The reason is that for non-convex regions, Voronoi division creates non-connected domains, and if the isolating proportion is large, the cells in non-connected domains are difficult to assign to the region where the agent’s initial position is located.

In our work, we re-examined the grid-based regional decomposition method and analyzed the cause of the “blind spot” where obtaining an effective sub-region division is difficult. We propose to use geodesic distance instead of general Euclidean distance, which enables faster convergence to a satisfactory solution for corner cases. Moreover, with a consideration for energy efficiency in practical applications, the number of turns in the path is effectively reduced by optimizing the shape of the sub-regions and the generation of the spanning tree, resulting in a more reasonable coverage for the target region.

## 3. Methods and Algorithms

In this paper, the term “coverage” implies that individual intelligences must actually visit the corresponding regions. An effective multi-agent coverage algorithm should generate a covering path for each agent, so that the union of all paths can produce full coverage of the region while minimizing the average waiting time. Initially, the target region is described based on a grid, and subregions are divided based on the number of agents and their initial positions. In subsequent stages, the exact path for each agent within its exclusive region is planned through a spanning tree.

### 3.1. Problem Description and Grid Representation

**Figure 1:**
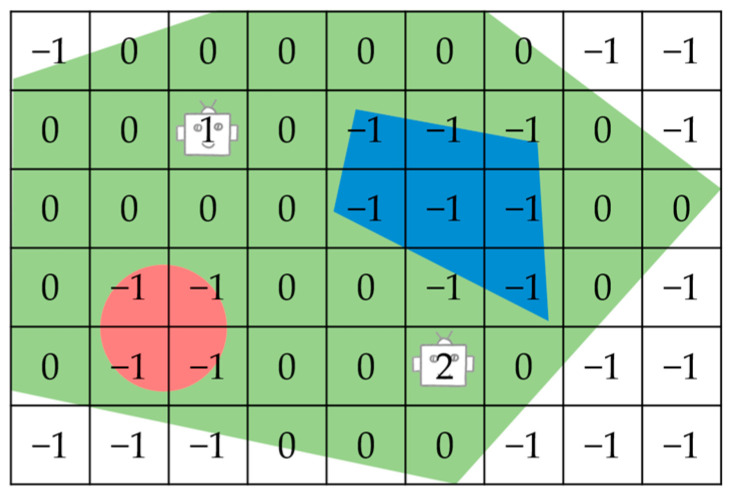
A tagged grid representation of the target region.

The discrete grid, due to its simplicity and flexibility, is one of the most commonly used forms of plane and surface representation in the field of computer graphics [35]. By gridding and discretizing the working area, it can simultaneously address the problems of obstacles and non-convexity in complex regions. As illustrated in Figure 1, a polygonal region to be covered is first represented using rectangles with coordinates (*x*, *y*), and then discretely represented as equal grid cells with a certain resolution. The centers of the cells are represented by nodes:

(1)  G={x, y:x∈[1, rows], y ∈[1, cols]}

The precision of grid discretization is dependent upon the scanning density $d_{s}$ of the region, which is expounded upon in detail in Section 3.3. Here, $d_{s}$ denotes the distance between two adjacent trajectories and is related to the range of sensing capabilities possessed by the agents. Using a smaller resolution in order to provide a more precise coverage will result in resource waste as it increases the time and energy requirements for task execution. In addition, for complete traversal of large-scale regions, it is necessary to describe them using grid maps of considerable size, which places great demands on memory and computational capacity. rows, cols are the number of rows and columns after discretization of the target region, then the number of rectangular cells used to expand the representation is n=rows×cols.

(2) $$\;rows=\frac{x_{max}-x_{min}}{d_{s}}$$

(3) $$cols=\frac{y_{max}-y_{min}}{d_{s}}$$

The target polygon and each obstacle can be described with a sequence of grid nodes to represent its boundaries:

(4) $$\;Polygon=\left\{\left(x_{1},\;y_{1}\right),\ldots ,\left(x_{n},\;y_{n}\right)\right\}$$

(5) $$\;Ob_{i}=(\left(x_{1},\;y_{1}\right),\ldots ,\left(x_{k},\;y_{k}\right),\;\forall i\;\in \left\{1,\ldots ,n_{ob}\right\}$$

A grid map can easily be represented as an array or matrix where each element expresses the occupancy status of a grid, including the location of the smart body, the status of its occupancy by obstacles, and whether it is a planable free grid. Define a rows×cols matrix GridEnv  that represents the occupancy information of the target area:

(6) $$\;GridEnv_{x,\;y}=\{\begin{array}{c} -1,\;If\;the\;unit\;is\;occupied\;\\ 0,\;If\;thecell\;is\;free\;\\ i,\;\forall i\;\in \left\{1,\ldots ,\;n_{r}\right\},\;Initial\;position\;of\;the\;agent\;i \end{array}$$

A complete representation of the tagged grid for the target region is shown in Figure 1. Within the graphic elements, green denotes the target area, red represents the no-entry zone, and blue signifies the obstacles. In utilizing the grid map description, the grid of the initial position of the agent is labeled as *i*. The explorable area is labeled as 0, while the remaining area is labeled as −1.

The set of free cells, excluding the cells outside the boundary and the cells occupied by obstacles, is defined as the explorable domain L.

**Definition** **1:** *Adjacent nodes* $\left(x_{i},\;y_{i}\right)$ *and* $\left(x_{j},\;y_{j}\right)$ *are defined as follows:*

(7) $$\;\|x_{i}-y_{j}\|+\|y_{i}-y_{j}\|\;\leq 1$$

This means that adjacent nodes must have common edges, which is in accordance with the physical constraints of the movement of the agents. Thus, the planned path for each agent is a sequence of adjacent cells:

(8) $$\;W_{i}=\left\{\left(x_{1},\;y_{1}\right),\ldots ,\left(x_{m_{i}},\;y_{m_{i}}\right)\right\}$$

where $m_{i}$ is the number of grid nodes on the path of the *i*-th agent. Furthermore, to ensure that the region can be explored cyclically, the planned path must be a closed path. In other words, $\left(x_{1},\;y_{1}\right)$ and $\left(x_{m_{i}},\;y_{m_{i}}\right)$ should be adjacent to each other.

We assume that the agents can accurately locate themselves within G, and at any time, it can move from its current grid cell to any unoccupied neighboring cell without any motion uncertainty. The position of the agents can be defined as:

(9) $$X_{i}\left(t\right)=\left(x_{i},\;y_{i}\right)\in L,\;\forall i\in \left\{1,\ldots ,\;n_{r}\right\}$$

where t denotes the time-stamp of the coverage path and $n_{r}$ denotes the number of multi-agents. The initial position of the *i*-th multi-agent is denoted as $X_{i}\left(t_{0}\right)$.

It has been noted that a multi-agent system does not yield N-fold productivity due to various factors. First, the spatial limitations of the environment can force the agents to move together. Second, mutual interference among multiple agents has been reported [5]. Third, an agent may explore areas that have already been searched by other agents without knowing their existence or location. Hence, the following conditions are defined as necessary to ensure an optimal solution for the mCPP:

1. All the paths of the agents should completely cover the region;
2. The area should be filled without overlapping paths, while avoiding all obstacles;
3. No preparation stage, and the agents can start traveling from their initial position;
4. Considering the constraints of the agents’ motion model, the trajectory should be simple and easy to control (e.g., straight lines or circles).

Combining the previous discussions, the grid-based mCPP problem can be described as follows:

Knowing the inputs Equations (1), (4), (5) and (9), plan the path $W_{i}\;,\;\forall i\in \left\{1,\ldots ,n_{r}\right\}$, such that

(10) $$\;\;\;\;\;\;\;\;\;\;\underset{W}{\min}{\;\max}_{\;i\;\in \left\{1,\ldots ,\;n_{r}\right\}}\left|W_{i}\right|\;\;\;\;\;\;\;\;\;\;s.t.\;\;\;W_{1}\cup^{\;}W_{2}\cup^{\;}\ldots \cup^{\;}W_{n_{r}}\;⊇L$$

where $\left|W_{i}\right|$ denotes the length of path $W_{i}$, i.e., the number of nodes in the sequence.

Given the aforementioned condition 1, if our strategy can further ensure that there is no overlapping between the paths of the agents, then in the region L, we only need to address the issue of establishing an $L_{i}$ region (without the requirement of convexity) for each agent. Hence, the problem can be further described as follows:

(11) $$\underset{W}{\min}{\;\max}_{\;i\;\in \left\{1,\;\ldots ,\;n_{r}\right\}}\left|L_{i}\right|\;s.t.\;\;\;L_{1}\cup^{\;}L_{2}\cup^{\;}\ldots \cup^{\;}L_{n_{r}}\;⊇L$$

where $L_{i}$ denotes the exploration region (not a strict path), which the agent $r_{i}$ is responsible for, $\left|L_{i}\right|$ is the number of grid cells in that region.

**Definition** **2:** *The equivalence conditions required to establish a sub-region optimal solution are summarized as follows:*

1.
$L_{i}\;\cap^{\;}\;L_{j}=\emptyset \;,\;\forall \;i\;,\;j\;\in \;1,\ldots ,n_{r}\;,i\;\neq \;j$ *;*
2.
$L_{1}\cup^{\;}L_{2}\cup^{\;}\ldots \cup^{\;}L_{n_{r}}\;⊇L$

3.
$L_{1}\approx L_{2}\approx \ldots \approx L_{n_{r}}$

4. $L_{i}$ *is connected* $\forall \;i\in \;1,\ldots ,n_{r}$
5.
$X_{i}\left(t_{0}\right)\in \;L_{i}$

The first condition ensures that the exploration paths among the agents do not overlap with each other, which is conducive to the generation of the optimal solution. The second condition ensures complete coverage of the free regions. The third condition ensures a balanced assignment of tasks, thus ensuring the minimization of the maximum waiting time among the agents. The fourth condition means that the assigned cells are directly accessible to the agents. This ensures that the exploration of sub-regions is a continuous and ordered operation, avoiding the additional cost of traversing non-connected regions (crossing non-connected regions requires consideration of the collision avoidance among agents, which generates another optimization problem). The last condition can ensure that there is no preparation stage for the agents to reach the responsible subregion before executing the exploration task.

### 3.2. Task Assignment

When deploying a set of agents, it is crucial to consider task assignment and coordination for enhancing system gain. Region division is the key to accomplish regional traversal using multiple agents, and it solves the mCPP problem by dividing it into several single-agent CPP problems. We continuously correct the distance matrix through an iterative process to construct an assignment matrix, labeling all cells to their corresponding unique agent. The feasibility of a balanced partitioning is explained in [36], where it is stated that any polygon (even non-convex) can be fairly divided into N pieces (i.e., each block has the same area, but is not necessarily convex).

For the agent i, the corresponding distance matrix $D_{i}$ records the travel cost of each cell in L. The assignment matrix A, which records the relationship between the grid and the agents, is obtained by combining $n_{r}$ distance matrices. During the construction process of A, each element is calculated by the following formula:

(12) $$\;A_{x,y}=arc\underset{i\;\in \left\{1,\;\ldots .\;n_{r}\right\}}{\min}D_{i\;|\;x,\;y}\;,\;\forall \;\left(x,y\right)\in L$$

That is, each cell is assigned to the agent with the lowest travel cost. Then the region $L_{i}$ of each agent can be calculated directly by the assignment matrix A.

(13) $$\;L_{i}=\left\{\left(x,y\right)\in L:A\left(x,y\right)=i\;\right\}\;,\;\forall \;i\;\in \left\{1,\ldots ,\;n_{r}\right\}$$

Adopting the above assignment strategy can fulfill the 1st, 2nd, and 5th conditions defined in Definition 2.

#### 3.2.1. The Balance of Regional Distribution

The distance matrix $D_{i}$ of the *i*-th agent records the distance between each cell in  L and the initial position $X_{i}\left(t_{0}\right)$ of the respective agent. If the distance function is selected as the Euclidean distance, the resulting assignment matrix A should be a classical Voronoi diagram. However, as is well-known, Voronoi partitions lack the principle of balance. Hence, we iteratively “correct” the travel costs of the distance matrices $D_{i}$ through the following formula, in order to fulfill condition 3 in Definition 2, ensuring the minimization of the maximum waiting time in the multi-agent system:

(14) $$\;D_{i}=m_{i}D_{i}$$

where $m_{i}$ is the iterative correction factor for the distance matrix of the *i*-th agent, and the update formula for each iteration is:

(15) $$m_{i}=m_{i}+\alpha \;\left(\left|L_{i}\right|-p_{i}\right),\;\overset{n_{r}}{\underset{i=1}{\sum }}p_{i}=1$$

where α is a positive adjustable parameter, and $p_{i}$ is the proportion of sub-region for the *i*-th agent. If each agent has the same exploration capability, then:

(16) $$\;p_{i}=\ldots =p_{n_{r}}=p$$

where $p=\left|L\right|/n_{r}$ represents the number of cells divided equally among L. From a formal perspective, the corrective effect of $m_{i}$ can be described as follows: when the responsible region of the *i*-th agent is smaller than the equilibrium share p, $m_{i}$ will decrease thus leading to a smaller path cost for the cells in $D_{i}$, which eventually results in more cells being assigned to the *i*-th agents during the construction of the assignment matrix A. Conversely, the same logic applies. The rigorous theoretical derivation of Equation (15) is described in detail in [8].

#### 3.2.2. The Connectivity of Regional Distribution

Since the target region is non-convex and there may be obstacles or no- entry areas inside, the above iterative procedure, although easier to converge, does not guarantee the continuity of the subregions (condition 3 in Definition 2). For the noncontiguous subregions, we introduced the correction matrix $T_{i}$:

(17) $$\;\;T_{i\;|\;x,y}=\frac{\beta \;\|\left[x,\;y\right]-X_{i}\left(t_{0}\right)\|}{1+\min\|\left[x,\;y\right]-q\|}\;,\;\forall q\;\in Q_{i}\;,\;Q_{i}=L_{i}\backslash R_{i}$$

where $R_{i}$ denotes the set connected to the initial position $X_{i}\left(t_{0}\right)$ in subregions $L_{i}$, while $Q_{i}$ denotes the set assigned to the i-th agent but not connected to $X_{i}\left(t_{0}\right)$. β is a positive adjustable parameter, and the presence of constant 1 in the denominator prevents $T_{i\;|\;x,y}$ from failing when [*x*, *y*] is in $Q_{i}$. The correction matrix $T_{i}$ serves to reward the region around the initial position $X_{i}\left(t_{0}\right)$ of the intelligence (reducing the path cost) and penalize the other region (increasing the path cost), gradually constructing a closed-shaped subregion. It is important to note that $T_{i}$ is constructed only when $L_{i}$ is a multiple disconnected domain.

Ultimately, to keep the assignment matrix A connected and balanced, the iterative equation for the distance matrix $D_{i}$ is

(18) $$\;D_{i}=T_{i}⊙(m_{i}D_{i})$$

**Figure 2:**
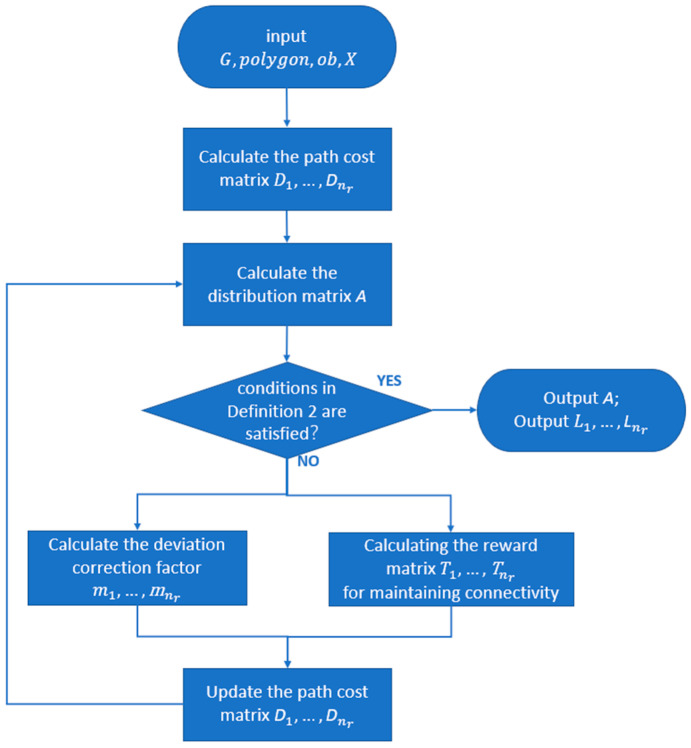
Flow chart of the algorithm.

In summary, the region partitioning algorithm uses the distance matrices from cells to each agent’s position as the measure. The results of the region allocation are continuously modified through a process of iterative correction factors to satisfy the conditions outlined in Definition 2. The correction factors include both balance correction factors and connectivity correction factors. The flow of sub-region assignment is shown in Figure 2.

**Figure 3:**
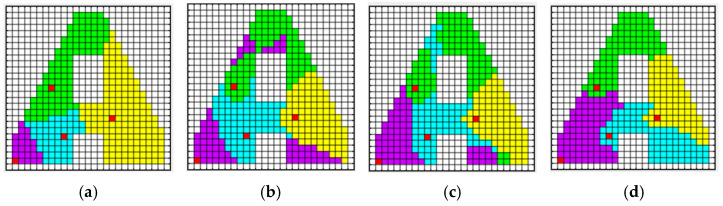
The result of sub-region assignment during the iteration. In the figure, the red grid represents the initial position of each agent, the white grid represents unexplored areas, and the other different colored grid domains represent the sub-regions of the target region. (**a**) Iteration = 0, (**b**) iteration = 160, (**c**) iteration = 320, (**d**) iteration = 480.

Figure 3 shows an execution example of a non-convex environment. The terrain consists of 50 × 50 cells, the number of agents $n_{r}$ is four, the initial positions of the agents are randomly distributed throughout the target area (indicated by the red), and each subplot illustrates the corresponding iteration situation of the assignment matrix A. Clearly, the algorithm is terminated after 480 iterations, satisfying all the conditions of Definition 2.

After sub-region assignment, each agent is assigned a specific part of the grid to cover. Exclusively sub-regions ensure non-intersecting paths, allowing multiple agents to operate safely and simultaneously while avoiding potential collisions among them.

The key feature of this algorithm allows the gradual incorporation of cells at any arbitrary location into the sub-regions. To be more specific, the algorithm temporarily violates the connectivity constraints of the assignment matrix in order to escape from local minima. Subsequently, the algorithm gradually eliminates the presence of unconnected regions and the connectivity of the sub-region $L_{i}$ is restored.

#### 3.2.3. Geodesic Distance

In Appendix A of [8], three intuitive cases of non-existence of optimal solution are mentioned. Additionally, we have also discovered that the proportion of isolation from obstacles also determines whether the assignment strategy can converge to an effective solution within limited iterations.

**Figure 4:**
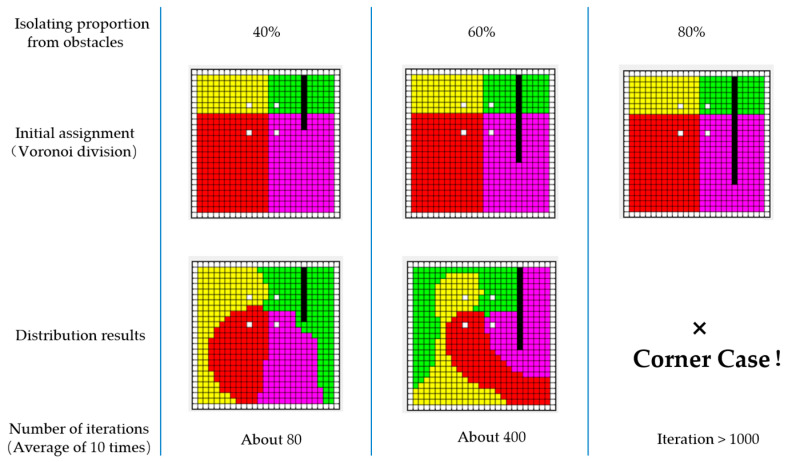
The proportion of partitions of barriers has an important impact on the regional distribution. In the figure, the four white grids represent the initial positions of the agents, the black grid represents the obstacle, and the other different colored grid domains represent the sub-regions of the target region.

As shown in Figure 4, when the proportion of isolation is relatively small (40%), the non-connected sub-regions (green) on both sides of the obstacle can quickly connect through the iteration Equation (15), thus converging to the goal defined in Definition 2. With the increase in the isolating proportion, the two non-connected regions become more difficult to connect. However, the process of global balanced assignment can be achieved by continuously expanding the set $R_{i}$ while reducing the set $Q_{i}$ to the empty set through the reward and penalty of matrix $T_{i}$. This process obviously has a greater number of iterations. Nevertheless, when the proportion of obstacle isolation increases to a certain extent, it becomes difficult to iterate to an effective solution.

We believe that the attribution of the initial grid has a crucial impact on the number of iterations. Taking the case of 80% isolating proportion in Figure 4 as an example, due to the strong isolating effect of obstacles, $Q_{i}$ in the upper right corner is almost unreachable to its attributed agent, thus the distance matrix $D_{i}$ cannot reflect the path cost. Therefore, we introduce a new distance representation, geodesic distance, to replace the original Euclidean distance.

The concept of geodesic distance originated from the field of geodesy in earth physics and is also referred to as geodesic or short-range line. It is mainly used to determine the local shortest path between two points in a curved space. In this paper, it is used to represent the cost of paths that bypass obstacles in a non-convex environment.

The geodesic algorithms on the surface include graph methods, partial differential equation methods, and optimization methods [35]. The division of the region in the previous description is actually the construction of a dense undirected graph, so graph search methods [37] can be directly used. Breadth-first search (BFS) is a search strategy for connected graphs, and the algorithm starts from a starting node “s” and successively searches for adjacent nodes. It radiates and traverses a wider surrounding area until it reaches the endpoint or the boundary (the maximum search depth).

**Figure 5:**
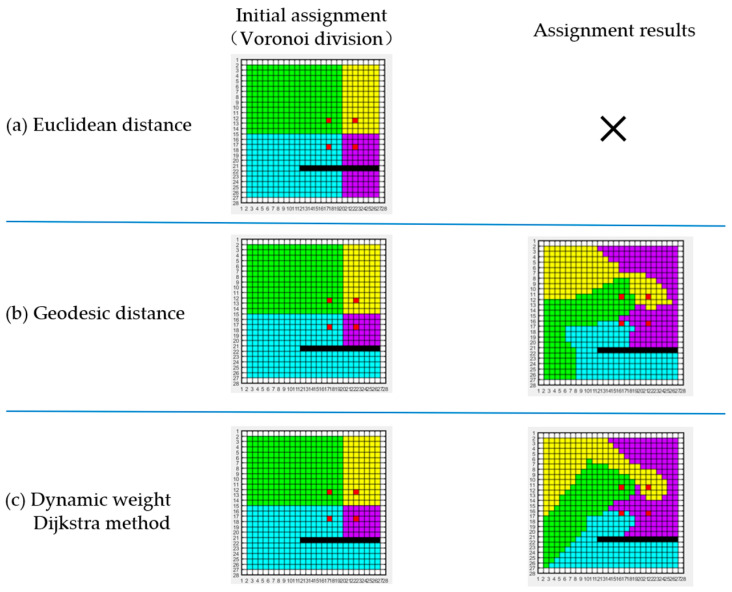
Comparison of the assignment results. In the figure, the red grid represents the initial position of each agent, the black grid represents the obstacle, and the other different colored grid domains represent the sub-regions of the target region. (**a**) Sub-region assignment using Euclidean distance. (**b**) Sub-region assignment using geodesic distance. (**c**) Dynamically assigning path cost weights by Dijkstra algorithms to make sub-region assignments more cohesive, thus suppressing the appearance of bridge-like narrow regions.

Figure 5 illustrates the region assignment for four agents during the execution time of the algorithm. Figure 5a shows the initial Voronoi division, while Figure 5b shows the assignment using geodesic distances, reaching convergence after only 300 iterations. The geodesic distances take into account the cost of paths due to obstacles, prohibited regions, and non-convexity of the boundaries in the environment. As a result, the initial partition of the target environment is reasonable, reflected by the absence of any disconnected sub-regions. It is worth noting that although the use of geodesics makes the initial division of all subregions connected, it is theoretically possible for a grid at any position to be assigned into any subregion during the iteration due to different values of “c”. That is, the sub-regions temporarily become non-connected during the iteration, so the correction matrix $T_{i}$ is still necessary and unavoidable.

The proposed algorithm primarily modifies its current state based on the globally optimal state, not solely by evaluating current information and candidate states. The algorithm approximates the behavior of gradient descent algorithms, possessing the capability of efficient search and reaching global optimality, even in the presence of multiple local minima.

#### 3.2.4. Geodesic Distance

The robustness and fault tolerance of the region coverage is highly unfavorable if there is a situation where sub-regions are connected by the width of only one or two cells. The “Future Work” section of [8] also mentions that if one or more constraints in the definition conditions are relaxed, the resulting subregions may be constructed to be only convex (less chaotic). However, in practice, it is difficult to guarantee the optimal solution if the conditions are relaxed, and the excessive demands on the shape will make the algorithm difficult to converge when the environment is complex.

Inspired by the Dijkstra algorithm [38], we enhance the coherence of the sub-regions by introducing suppression weights into the search to avoid the appearance of bridge-like narrow areas, as shown in Figure 5c above. The optimized shape will provide the agents with better fault tolerance during exploration.

**Figure 6:**
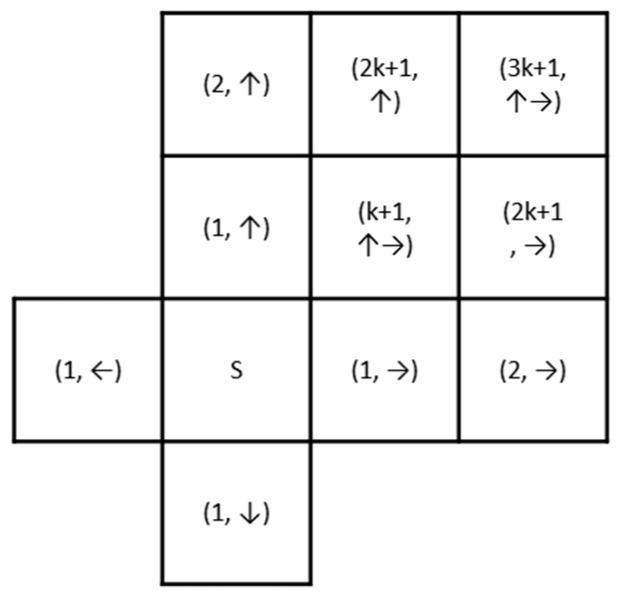
Calculation schematic of expanding the search from the origin to the surrounding cells. The arrow indicates the direction from the “parent node” to the current node. The shortest path tree is dynamically weighted based on Dijkstra’s algorithm with the change of search direction.

However, it is noteworthy that, unlike the Dijkstra algorithm, which searches based on known global cost, this paper assigns weights to the path cost dynamically and locally. As shown in Figure 6, at each step of the grid search, the source direction from the “parent node” to the current node is recorded. When the search direction changes, a path cost less than 1 is assigned to suppress the horizontal and vertical expansion from the source node “s”. The range of the path cost “k+1” for turning should be within the range of Manhattan distance and Euclidean distance, i.e.,$\;\sqrt{2}<k+1<2$.

The improved Dijkstra algorithm replaces BFS, and dynamically introduces the turning weight to calculate the shortest path tree. The specific steps are shown in Algorithm A1 in Appendix A. If the grid representation of the environment is considered as an undirected graph, the number of grid nodes is n=rows×cols and the number of edges is m=2rows×cols−(rows+cols). The complexity of this algorithm, when using the calculation method of maintaining arrays, is $O\left(m+n^{2}\right)=O\left(n^{2}\right)$. When using the priority queue data structure, the time complexity can be reduced to O((m+n)logn). When using the Fibonacci heap, the average cost of the relaxation operation is O(1), and the time complexity can be further optimized to O(m+nlogn).

### 3.3. Path Planning for Sub-Regions

Applying the assignment algorithm in Section 3.2, the target region is divided into multiple balanced and connected sub-regions. The original multi-agent optimization problem is reduced to $n_{r}$ single-agent CPP problems, and now we only need to consider the minimum coverage time of each agent within its own region:

(19) $$\underset{W_{i}}{\min}\;\left|W_{i}\right|\;\;s.t.W_{i}\;⊇L$$

**Figure 7:**
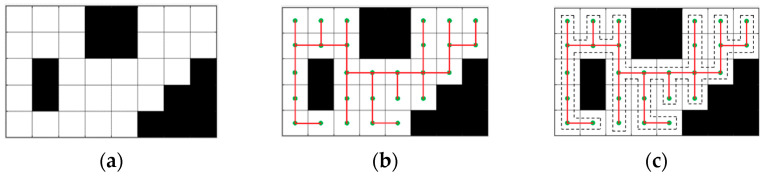
Basic steps to design a sub-region coverage path. The green points represent the center of each grid and serve as the nodes used to construct the tree. The red lines represent the constructed tree. (**a**) Representation of grid discretization. (**b**) Construction of a spanning tree with each cell as a node. (**c**) Construction of a closed path around the spanning tree.

A spanning tree path covering method with complexity O(n) is given in [39]. It starts from any unoccupied unit and constructs a spanning tree that covers all the target region cells. Subsequently, a coverage path is generated around it. Figure 7 shows the basic steps of trajectory construction through an example. The white grids represent the explorable free area, whereas the black grids indicate the obstructed area. A spanning tree is constructed using the nodes at the center of the free grids, while the dashed line represents a closed path surrounding the constructed spanning tree.

In this method, the target area is discretized into square cells of size $2d_{s}$. Each cell is either completely occupied by obstacles or no-entry zones, or is a completely free cell (refer to Figure 7a). A minimum spanning tree is constructed using any algorithm such as DFS, Kruskal, or Prim algorithms, with each free cell being a node and adjacent free cells being connecting edges (refer to Figure 7b). Then, around the tree, a closed path for the agent is constructed among adjacent cells of size $2d_{s}$ in a clockwise (counter-clockwise) direction, forming a complete coverage path with a scanning density of $2d_{s}$ (refer to Figure 7c).

Additionally, the number of turns is one of the major factors affecting travel time and energy consumption. It is believed that the choice of the initial spanning tree has a profound impact on coverage efficiency. If the shape of the spanning tree can be properly constructed to minimize turns in the in the closed path of the agent, it can greatly reduce the coverage time in practical applications. To address this issue, this paper appropriately assigns weights to edges in the grid undirected graph and uses this feature to control the connection of nodes in each sub-region’s spanning tree, generating a spanning tree shape that is inclined to follow a specific coordinate direction, thereby affecting the shape of the coverage path.

**Figure 8:**
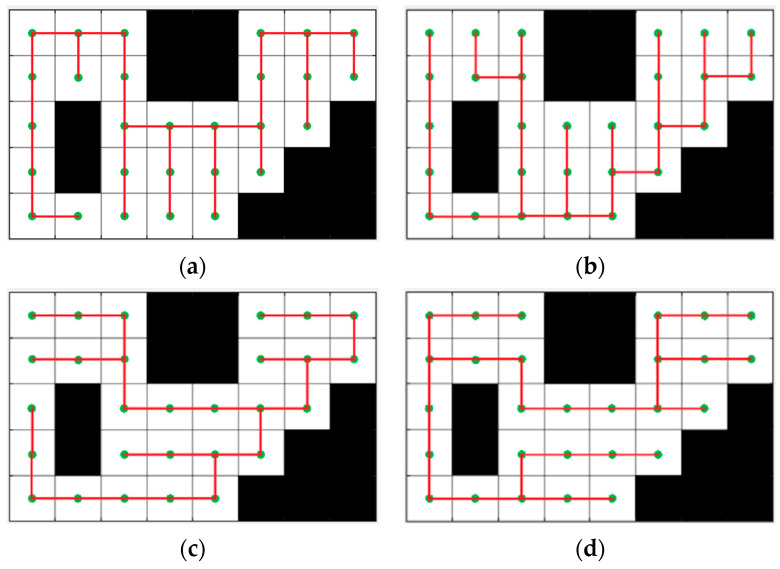
Spanning tree shapes with different weight combinations. (**a**) Spanning tree shape of up mode. (**b**) Spanning tree shape of down mode. (**c**) Spanning tree shape of right mode. (**d**) Spanning tree shape of left mode.

Table 2 shows the selected combination of weights for horizontal and vertical edges, where i is the row number of the grid graph and j is the column number of the grid graph. Figure 8 shows the path planning results under different weight combinations and provides an illustration of the impact on the overall system performance. Evidently, in the same operational environment, the “left” mode requires only 31 turns to completely cover the region of interest, which reduces 18% compared to the “up” mode. Of course, this method yields better optimization results for environments with greater aspect ratio differences.

### 3.4. Overview of the Proposed Algorithm

The mCPP algorithm proposed in this paper solves the problem of planning paths for multi-agents that participate in a complete traversal of the region, given:

- The user-defined region of interest, Polygon;
- The sub-regions of the polygon representing obstacles/no-entry zones, $Ob_{1},Ob_{2},\ldots ,Ob_{j},\ldots$;
- The required scan density $d_{s}$ for path coverage;
- The number of agents $\;n_{r}$, and their assignment proportion $p_{i}$;
- The initial positions $X_{1}\left(t_{0}\right),\ldots ,X_{n_{r}}\left(t_{0}\right)$ of each agent in the target region.

As the output of the proposed algorithm, the corresponding coverage path $W_{i}$ for each agent is obtained. The computation process of the algorithm integrates two stages, the exploration task balance partitioning and the spanning tree planning of sub-regions. In the first stage, the region partitioning algorithm divides the exploration domain L into $n_{r}$ exclusive regions $L_{i}$, as the independent operation domains for each agent. Hence, the original multi-agent optimization problem Equation (10) is degraded to $n_{r}$ single-agent CPP problems, alleviating its explosive combinatorial complexity. Each of these optimization problems can be represented as Equation (19), which utilizes a spanning-tree-based path planning algorithm to compute the closed loop path in each sub-region. The result ensures continuous exploration and repeated visits to each node of the target region.

It should be noted that although the final paths $\left\{W_{1},W_{2},\ldots ,W_{n_{r}}\right\}$ are constructed in a fully distributed manner, the resulting overall solution W does not compromise in terms of effectiveness and generality in solving problem Equation (10). And the algorithm fully satisfies all the requirements of the optimal solution that we proposed for this problem in Section 3.1.

Recursive algorithms may have certain limitations and potential challenges in highly complex regions. First, recursive algorithms may be affected by computational resource constraints, as new memory space needs to be constantly created and freed during the recursion process, which requires the processing power and storage capacity of the computer. Additionally, the recursive algorithm may not perform well in some highly nonlinear and non-stationary problems, and may encounter problems such as numerical instability. To address these limitations and potential challenges, we can consider expanding or replacing the recursive algorithm with other methods. For example, more efficient optimization algorithms such as deep learning can be explored to better handle highly complex problems. Moreover, different decomposition and merging strategies can be explored to enhance the robustness and reliability of the algorithm.

## 4. Results and Discussion

**Figure 9:**
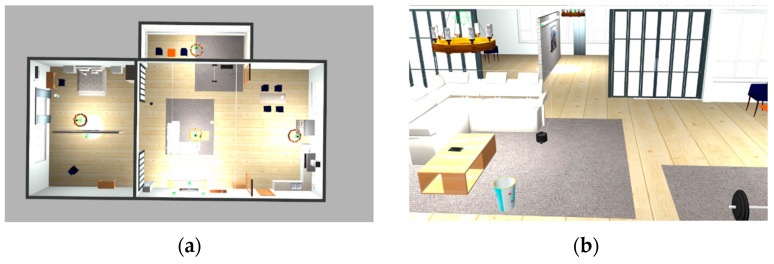
Example: Path coverage process in indoor environment. (**a**) Modeling of the interior environment in Gazebo. (**b**) Modeling details.

In the previous analysis, we have considered differently shaped regions, including concave polygon (Figure 3) and rectangles with high barrier-to-space ratio (Figure 5), which intuitively demonstrate the effectiveness and robustness of our algorithm. Next, we consider a real-world indoor scenario for path coverage task. Figure 9 show the modeling of an indoor environment, which is overall a convex polygon. The interior of the region contains holes caused by furniture arrangements, as well as large-scale separations of the walls to the space.

**Figure 10:**
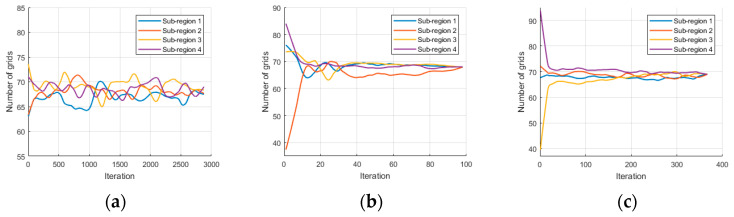
Iterative process for sub-region assignment. (**a**) The original algorithm while the span to smooth is 200. (**b**) Iterative process using geodetic distance while the span to smooth is 12. (**c**) Iterative process with optimization of regional cohesion while the span to smooth is 35.

Here, the mCPP process for the given area with 4 agents was analyzed. The iteration process for subregion assignment was plotted in Figure 10. Due to the existence of fluctuations, a smoothing process was performed during the plotting process. The specific method is local regression using weighted linear least squares and a first-degree polynomial model, and the span for calculating the smoothed value is determined by the number of iterations.

Figure 10a shows the result of the original algorithm, and it can be observed that after 2900 iterations, the balance condition for subregion assignment was not yet satisfied. The original algorithm fails in complex indoor environments with many obstacles and large wall separations. Figure 10b presents the iteration process using geodesic distance, and it can be seen that the balance condition is satisfied after the 97th iteration, achieving convergence very fast. Figure 10c shows the iteration process with shape optimization added, which converged after 367 iterations. Specific evaluation metrics and their improvement effects will be further analyzed in the following sections.

**Figure 11:**
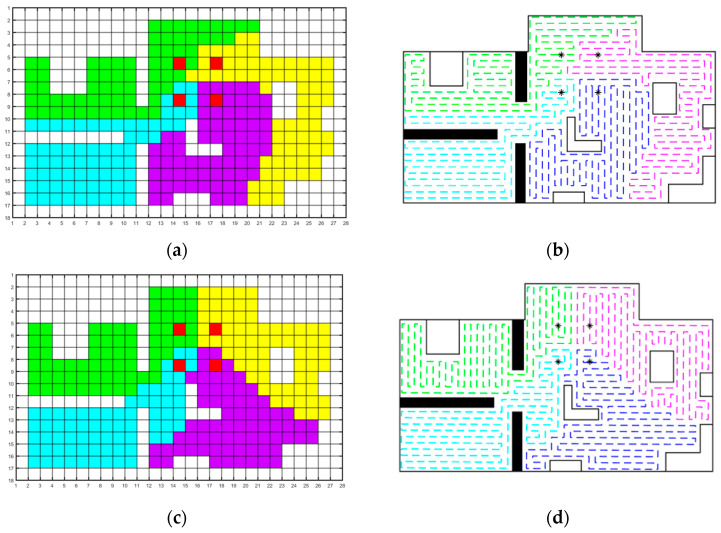
The planning process of the algorithm. The red grid and “*” denote the initial position of each agents. The grids of different colors represent the sub-regional divisions corresponding to different agents, while the dashed line represents the corresponding scanning path. (**a**) Results of sub-region assignment using geodetic distance. (**b**) The results of multi-agent path planning using geodetic distance. (**c**) Results of sub-region assignment by optimization of regional cohesion. (**d**) The results of multi-agent path planning by optimization of regional cohesion.

The path coverage planning for the indoor region is performed with four agents, and the process is shown in Figure 11. The non-convex target domain is divided into balanced sub-regions considering the initial distribution of multiple agents. Within each agent’s subregion, a closed coverage path is planned, ensuring that each node is covered with the same optimal frequency. However, after dynamic weighting for shape optimization, the sub-regions become more cohesive and regular. The specific evaluation metrics and their improvement effects will be further analyzed in the following statement.

**Figure 12:**
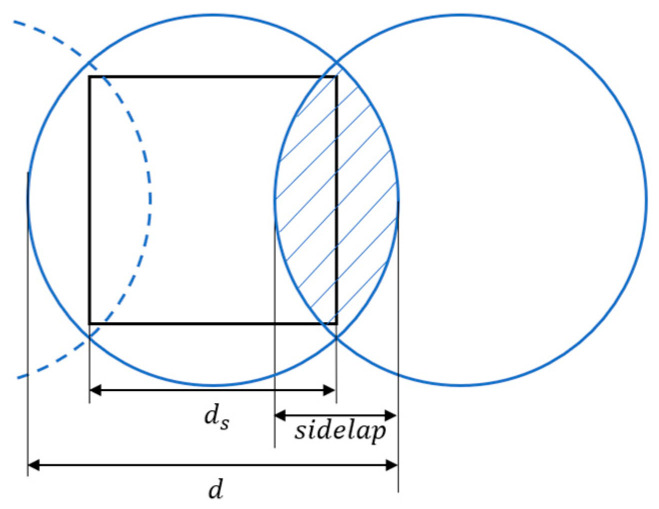
Schematic diagram of sensing range overlap for adjacent paths.

In practical applications, the detection range of sensors carried by the agents is typically circular, with a coverage diameter of d. As shown in Figure 12, there is overlap of information in the sensing range of adjacent path nodes, where the overlap length is referred to as sidelap. Considering the overlapping of adjacent sensors, the overlap percentage p is defined as:

(20) $$\;p=\frac{2\times sidelap}{d}$$

In general, the terms d, $d_{s}$, sidelap, and p have the following relationships:

(21) $$d_{s}=d-sidelap=d\left(1-\frac{p}{2}\right)$$

**Figure 13:**
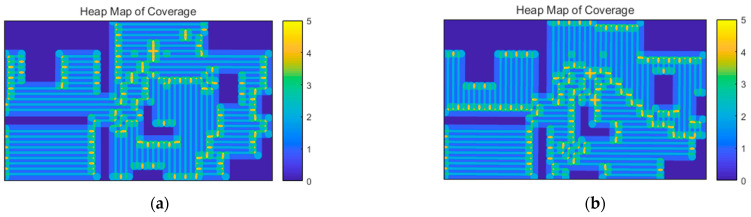
Heat map for indoor coverage tasks. (**a**) Using geodetic distance. (**b**) Adding optimization of regional cohesion.

Considering the sensing redundancy of each cell, there should be $d>\sqrt{2}d_{s}$. That is, the sensing diameter should be greater than the diagonal length of the cell formed by the scanning density $d_{s}$. The generated coverage paths are simulated by selecting $d=1.5\;d_{s}$, i.e., p=66.6%. Considering the number of scans performed at various locations within the region, the coverage heatmap of the target area is shown in Figure 13.

Table 3 explains the performance metrics used to evaluate the effectiveness of the coverage task. Unlike in Section 3.2.3, where we primarily focus on the number of iterations for region division, here we quantitatively evaluate the features of the generated path.

The original algorithm and the proposed optimization solution are quantified using the performance metrics listed in Table 3. The comparison results are shown in Table 4. It can be seen that, for non-convex regions with many holes and a higher isolating proportion, such as the region in Figure 10, the original algorithm is unable to effectively assign the region through efficient iteration without the proposed optimization.

The optimization scheme has the same index of coverage (PoC) since the grid description of the target region is the same. The optimization scheme dynamically assigns weights to the path cost by an improved Dijkstra algorithm, making the sub-region assignment more reasonable (increased cohesiveness). Combined with the selection of different weighting schemes for the spanning tree, the number of turns in the coverage paths and the repeated sensing regions are both significantly reduced, resulting in an improvement in all types of metrics. To be more precise, the sub-region cohesion optimization method reduced the number of turns by 22.85%, the percentage of overlapping decreased by 22.17%, significantly reducing the wasted resource of redundant scanning in the execution of the operation.

## 5. Conclusions and Future Work

When using multiple agents for cyclic exploration of a target region, it is important and critical to balance the assignment of tasks for each agent and to plan the corresponding paths within their respective regions. In this paper, we investigate the algorithms related to the grid description of the environment, sub-region assignment and shape optimization, and path planning within a subregion, and the main results and innovations achieved can be summarized as follows:

(1) In terms of region assignment, we introduce geodesic distances to cope with the problem that Euclidean distances are not directly accessible in non-convex regions. This method optimizes the Voronoi partition for the initial state and is effective for narrow regions and near-closed regions. In addition, we noticed that the partitioned sub-regions may have narrow “bridge” areas, due to the initial positions of agents and the obstructing effect of obstacles. It can be detrimental to the stability of the exploration process if the connectivity of the sub-regions is too dependent on some “bridge” cells. Therefore, this paper also optimizes the shape of sub-regions through a modified Dijkstra method to assign weights to step distances. So that the grid attribution is more reasonable and the robustness of the mCPP strategy is stronger. It is worth noting that the more “cohesive” sub-region division can plan paths with fewer turns and save turning energy.
(2) For path planning, we describe the RRT algorithm for sub-region coverage paths. By appropriately assigning weights to edges in the grid graph, the shape of the generated tree is appropriately constructed, thereby affecting the shape of the coverage path, minimizing the turns in the closed-loop path of the agent.
(3) In addition, we have built a simulation experimental platform for multi-robot systems in Gazebo and performed the application of the scheme in non-convex scenarios with indoor holes and strong partitions.

The algorithm of regional path coverage proposed in this paper has been verified by simulation experiments to be feasible and provides new ideas and tools for the engineering field. There are still some problems worthy of further exploration, and future research work can be carried out in the following directions.

(1) As the area to be covered grows linearly, the memory required by the grid-based method grows exponentially in finding the optimal paths, which is not suitable for large areas with small sensing ranges [9]. Grid-based coverage methods are therefore suitable for mobile robot operations in more complex indoor environments, since the area to be covered is usually relatively small and the representation of local occupancy information is more flexible. For large or fine-resolution grids, on the one hand, a cell decomposition approach such as the boustrophedon decomposition [40] can be used, but it is also important to consider that covering individual cells becomes more complex; on the other hand, a layered approach can also be considered for the representation of vast free areas and complex areas that need to be finely described in different resolutions.
(2) In the process of region division, geodesic distance is introduced to express the path cost in non-convex environments. In fact, geodesic line is a highly intrinsic quantity on a surface and has far-reaching significance in fields such as computational geometry and industrial production. Therefore, we may attempt to extend our task to surfaces (or complex terrains) to meet the demands of applications such as 3D scanning.
(3) According to Galceran [10], the mCPP strategies can be classified as offline or online. The offline algorithms only rely on static information and assume that the environment is a priori known, which may not be unrealistic in many cases. The online algorithms do not assume prior knowledge of the environment, instead relying on real-time sensor measurements to scan the target space [24]. In our future plan, we plan to develop an online version of the multi-agent completely coverage path planning algorithm, so that it can operate in a completely unknown area based on sensors. The agents will be able to switch or take over other areas as needed.

## Figures and Tables

**Table 1:** Overview of recent mCPP work.

Method	Characteristics	Task Balance	Closed Path	No Preparation	Corner Case (Extremely Narrow)	Application Validation
[25]	Non-convex	√	×	×	×	×
[26]	Non-convex	×	×	√	×	×
[27]	Convex	√	×	×	×	×
[4,6,7]	Grid with obstacles	× *	√	√	√ *	×
[8,28]	Grid with obstacles	√	√	√	×	×
Proposed	Grid with obstacles	√	√	√	√	√

* Not explicitly mentioned in the papers, but the feature is supported inherently by the method used.

**Table 2:** Combination of weights of horizontal and vertical edges in different modes.

Mode	Horizontal Edges	Vertical Edges	Number of Turns (Figure 7)
up	rows−i	1	38
down	1+i	1	40
right	1	cols−j	36
left	1	j+1	31

**Table 3:** Path evaluation indicators and its meaning.

Indicator	Explanation	Unit
PoC	Percentage of Coverage (Scans ≥ 1)	%
PoOC	Percentage of Overlapping Coverage (Scans ≥ 2)	%
Turns	Number of Turns	-
N-Turns	Number of turns after normalization	$\frac{Turns}{d_{s}{}^{2}}$

**Table 4:** Comparison of evaluation indicators between the original algorithm and the improved solutions.

Indicator	Original Algorithm	Use of Geodetic Distance	Optimization of Regional Cohesion
PoC	failed	98.53%	97.74%
PoOC		67.46%	56.39%
Turns		279	233
N-Turns		17.42	14.56

## Data Availability

The data used to support the findings of this study are available from the corresponding author upon request.

## Appendix A

The following algorithm is used to calculate the shortest path tree by the improved Dijkstra algorithm, where *typeA* denotes the nodes for which the shortest path has been found; *typeB* denotes the candidate nodes in the container, i.e., the first-order neighborhood nodes of the *typeA* nodes; and *typeC* denotes the nodes that have not yet been visited, including the free cells and the cells where agents are located.

**Algorithm A1:** Calculate shortest path tree using improved Dijkstra algorithm
Input: Occupancy information: GridEnv; Initial position: $\;s=X_{i}\left(t_{0}\right)$ Output: The path cost matrix of the agent: Dis 1 foreach g∈G do: 2 $D_{i\;|\;x,y}\;:=MAX\_VALUE$ 3 $Dirction_{i\;|\;x,y}\;:=0$ 4 Dis(s) :=0; Dir(s) :=0 5 GridEnv(s) :=typeB 6 Q←s 7 while (Q ≠ ∅) do: 8 cur :=Extract_Min(Q) 9 GridEnv(cur) :=typeA 10 $foreach\;next\in cur^{’}s\;neighbors\;\&\;next\in L$ do: 11 if Dir(next)== Dir(cur) do: 12 Dis_temp=Dis(cur)+1.0 13 else do: 14 Dis_temp=Dis(cur)+k 15 if Dis_temp<Dis(next) do: 16 Dis(next)=Dis_temp 17 Dir(next)+=(cur→next) 18 if GridEnv(next)==typeC do: 19 Q ←next 20 GridEnv(next):=typeB 21 return Dis
